# Active Stratification of Colloidal Mixtures for Asymmetric Multilayers

**DOI:** 10.1002/smll.202404348

**Published:** 2024-08-16

**Authors:** Baekmin Q. Kim, Jongmin Q. Kim, Hojoon Yoon, EunSuk Lee, Siyoung Q. Choi, KyuHan Kim

**Affiliations:** ^1^ Department of Chemical and Biomolecular Engineering Korea Advanced Institute of Science and Technology (KAIST) Daejeon 34141 Republic of Korea; ^2^ Interface Materials and Chemical Engineering Research Center Korea Research Institute of Chemical Technology (KRICT) Daejeon 34114 Republic of Korea; ^3^ Department of Chemical and Biomolecular Engineering Seoul National University of Science and Technology (SeoulTech) Seoul 01811 Republic of Korea; ^4^ KAIST Institute for the Nanocentury KAIST Daejeon 34141 Republic of Korea

**Keywords:** colloidal particles, depletion pressure, functional films, interfaces, interfacial activity, multilayers, stratification

## Abstract

Stratified films offer high performance and multifunctionality, yet achieving fully stratified films remains a challenge. The layer‐by‐layer method, involving the sequential deposition of each layer, has been commonly utilized for stratified film fabrication. However, this approach is time‐consuming, labor‐intensive, and prone to leaving defects within the film. Alternatively, the self‐stratification process exploiting a drying binary colloidal mixture is intensively developed recently, but it relies on strict operating conditions, typically yielding a heterogeneous interlayer. In this study, an active interfacial stratification process for creating completely stratified nanoparticle (NP) films is introduced. The technique leverages NPs with varying interfacial activity at the air–water interface. With the help of depletion pressure, the lateral compression of NP mixtures at the interface induces individual desorption of less interfacial active NPs into the subphase, while more interfacial active NPs remain at the interface. This simple compression leads to nearly perfect stratified NP films with controllability, universality, and scalability. Combined with a solvent annealing process, the active stratification process enables the fabrication of stratified films comprising a polymeric layer atop a NP layer. This work provides insightful implications for designing drug encapsulation and controlled release, as well as manufacturing transparent and flexible electrodes.

## Graphical abstract

Colloidal mixtures are stratified at the air–water interface depending on their interfacial activity, forming asymmetric multilayers. With the help of depletion pressure, lateral compression induces individual desorption of less interfacial active nanoparticles (NPs) into the subphase, while more interfacial active NPs remain at the interface. This active stratification process leads to nearly perfect stratified NP films with controllability, universality, and scalability.

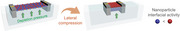

## 1 Introduction

Stratified films, composed of two or more layers of diverse components, have garnered significant attention across various applications including membranes,^[^ ^1^, ^2^ ^]^ coatings,^[^ ^3^, ^4^ ^]^ photovoltaics,^[^ ^5^ ^]^ electronics,^[^ ^6^ ^]^ and biomedical engineering,^[^ ^7^, ^8^ ^]^ due to the synergistic effects between each layer that lead to advanced performance and multifunctionality. Specifically, stratified films find direct applications in energy‐related fields, for example, energy conversion devices utilizing stratified asymmetric nanoparticle (NP) multilayers^[^ ^9^ ^]^ or transparent and flexible electrodes comprising a polymer thin film on top of a colloidal particle layer.^[^ ^10^ ^]^ It is most crucial to effectively manufacture fully stratified asymmetric multilayers for imparting excellent performance and functionalities in the applications.

Over the past few decades, various approaches have been developed to successfully implement stratified films, with the layer‐by‐layer (LBL) method being the most widely used and representative.^[^ ^11^, ^12^, ^13^ ^]^ The LBL method involves sequential deposition of each layer using common coating techniques, such as spin‐coating, dip‐coating, electromagnetic‐coating, and spray‐coating, etc., thereby resulting in structures with completely separate layers.^[^ ^11^, ^13^ ^]^ While this method is conceptually simple, in practice it is time consuming, labor intensive, and prone to in‐film defects, due to the iterative process.^[^ ^14^, ^15^, ^16^ ^]^ Accordingly, self‐stratification approaches, which enable the spontaneous stratification of components in a one‐step procedure, have emerged as compelling alternatives.

In conventional self‐stratification processes, blends or mixture solutions of thermodynamically incompatible polymers are stratified during melting or solvent evaporation, respectively, driven by differences in surface tension, substrate wetting, or contraction force, etc.^[^ ^14^, ^17^, ^18^ ^]^ However, only some specific pairs of polymers are applicable to these processes,^[^ ^14^, ^17^, ^18^ ^]^ and even the use of organic solvents raises environmental concerns.^[^ ^19^ ^]^ Against this background, attention has turned to the self‐stratification process exploiting drying binary colloidal mixtures (e.g., latex and inorganic particles), because water can be used as a medium and a wide range of particle combinations is applicable.^[^ ^15^, ^16^, ^20^, ^21^, ^22^, ^23^ ^]^ The distribution of particles in a drying colloidal film is mainly determined by two competing factors, particle diffusion and solvent evaporation, and is indicated by the dimensionless Péclet number (*Pe* = evaporation rate/diffusion rate): When *Pe* << 1, particles diffuse out uniformly, whereas when *Pe* >> 1, they accumulate near the top of a drying film. The difference in *Pe* between particles and the interaction between particles determine whether particles are stratified and the composition of the stratified film,^[^ ^15^, ^16^, ^20^, ^21^, ^22^, ^23^ ^]^ and this has also been extended to colloid‐polymer systems.^[^ ^24^, ^25^, ^26^ ^]^ Despite the great advantages, the self‐stratification process exploiting drying binary colloidal mixtures still has several drawbacks. It requires differently sized particles for *Pe* variation and demands precise control of operating conditions, such as diffusion rate, evaporation rate, and particle concentration, etc., complicating process design. Furthermore, since the stratification is based on the distribution gradient of particles rather than active segregation, it results in a heterogeneous interlayer that is not completely segregated, weakening film performances.^[^ ^15^, ^16^, ^20^, ^21^, ^22^, ^23^ ^]^ Hence, it is imperative to develop a new process that produces fully stratified films without requiring complex operating conditions.

**Figure 1:**
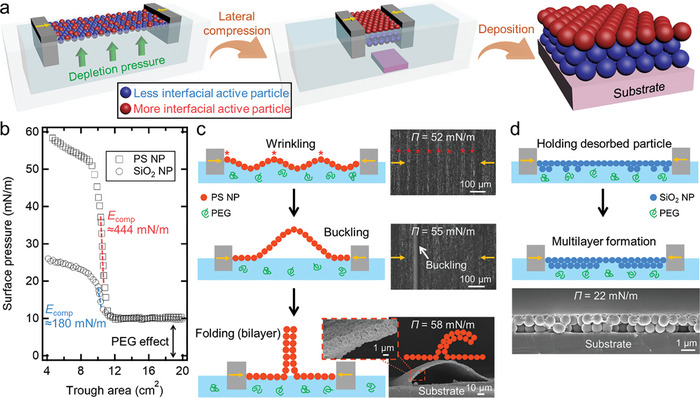
Concept of the active stratification process proposed in this work. a) Schematic illustration of the active stratification process proposed in this work. b) Compressional Langmuir isotherms of the PS (diameter = 960 nm) and SiO_2_ (diameter = 700 nm) NP monolayers at the air–water (containing 0.8 wt% PEG) interface. The SiO_2_ and PS NP monolayers withstand *Π* up to ≈20 and ≈50 mN m^−1^, respectively, and then collapse by further compression. The compressional modulus (*E*_comp_) is calculated as *E*_comp_ = ‐*A*·(d*Π*/d*A*), where *A* is the trough area, when the close‐packed monolayers are compressed (indicated by the dashed lines). c,d) Schematic illustrations and corresponding scanning electron microscope (SEM) or optical microscope images of the collapsing PS NP monolayer c) and SiO_2_ NP monolayer d) according to lateral compression at the interface. The collapsing PS NP monolayer exhibits out‐of‐plane deformations in the order of wrinkling, buckling, and folding as compression proceeds. The images of the wrinkling and buckling are top‐view optical microscope images, and the image of the folding is a cross‐sectional SEM image. The asterisk markers indicate the crest points of the wrinkles. The SEM image shows the cross‐section of the SiO_2_ NP bilayer that results from the collapsing SiO_2_ NP monolayer at the interface.

In this study, we introduce an active stratification process for manufacturing fully stratified NP films at the air–water interface. Recently, fluid–fluid interfaces (e.g., liquid–liquid and air–liquid interfaces) have served as effective 2D scaffolds for crafting intricately structured thin films.^[^ ^27^, ^28^, ^29^, ^30^, ^31^, ^32^ ^]^ In our proposed active stratification process utilizing the air–water interface, the only conditions to be considered are the difference in interfacial activity of NPs and the presence of depletion pressure (*P*_de_). As schematically illustrated in **Figure** **1**a, the active stratification process encompasses three key steps: 1) A mixture of NPs with different interfacial activity is spread as a monolayer at the air–water interface; *P*_de_ between the interface and the less interfacial active NP suppresses its desorption from the interface;^[^ ^29^, ^33^ ^]^ 2) When the initial monolayer is collapsed by lateral compression, only the less interfacial active NPs are actively pushed out of the interface into water but are held underneath the residual layer of more interfacial active NPs, thanks to *P*_de_;^[^ ^29^, ^33^, ^34^ ^]^ 3) The stratified NP film at the interface is deposited onto a solid substrate by the inverted Langmuir–Schaefer technique.^[^ ^35^ ^]^ Depending on the ratio of the interfacial area occupied by each type of NP and the degree of compression, the number of layers and the composition of each layer can be finely tuned. Moreover, the active stratification process facilitates the straightforward creation of fully asymmetric multilayers, wherein the top layer comprises solely conductive nanowires. Additionally, when complemented by a solvent annealing process, it enables the fabrication of layered films comprising a polymeric layer atop a NP layer. This greatly enhances the potential for applying this technology to functional films, including transparent and flexible electrodes.

## 2 Results and Discussion

### 2.1 Distinctive Collapse Behaviors of Each NP Monolayer

We utilize polyethylene glycol (PEG) as a depletant and employ polystyrene (PS) NP with a diameter of 960 nm and SiO_2_ NP with a diameter of 700 nm as a representative NP pair with different interfacial activity. We choose 35 000 g mol⁻¹ as the molecular weight of PEG for exerting effective *P*_de_ on the system based on our previous studies.^[^ ^29^, ^33^ ^]^ The PS NP demonstrates considerably higher interfacial activity, compared to that of the SiO_2_ NP. Upon introducing a suspension of each NP onto the air–water (containing 0.8 wt% PEG) interface, the NPs adsorb to the interface and gradually approach one another under lateral compression at the interface, ultimately forming a well‐packed NP monolayer.^[^ ^29^, ^35^ ^]^ As shown in Figure 1b, these well‐packed NP monolayers correspond to sharp increases in surface pressure (*Π*), and upon further compression, an abrupt change in the *Π* slope is observed, signifying the onset of collapse. The initial *Π* (≈10 mN m^−1^) is attributed to the interfacial energy decrease provided by PEG molecules as they are being adsorbed at the interface. The collapse behaviors of NP monolayers significantly vary depending on the interfacial activity of NPs,^[^ ^29^, ^35^, ^36^, ^37^, ^38^ ^]^ and this can act as a driving force for the complete stratification of NP mixtures at the interface. Hence, the selection of NPs with markedly different collapse behaviors is imperative, and an effective approach to this is to compare the compressional Langmuir isotherms of each NP.

For PS particles, previous studies have reported that the contact angle of water (*θ*_wca_) on the particle surface is ≈90°, irrespective of PEG adsorption on the surface.^[^ ^39^, ^40^ ^]^ Based on the balance between the adsorption energy of the PS NP to the air–water interface and the energy exerted by lateral compression, the maximum *Π* (*Π*_max_) applicable to the PS NP monolayer at the interface is estimated to be approximately equal to the interfacial tension (*γ*), in both the presence and absence of PEG (for more details, see Discussion S1, Supporting Information). The theoretical estimation suggests that *Π*_max_ is ≈72 mN m^−1^ in the absence of PEG and ≈62 mN m^−1^ in the presence of PEG, respectively, and is well agreed with the results from the compressional Langmuir isotherms. As shown in Figure 1b; and Figure S1a (Supporting Information), the PS NP monolayers withstand *Π* up to ≈50 mN m^−1^, and this is slightly lower than the estimated values due to the friction between the NPs during compression.^[^ ^35^, ^37^ ^]^ With further compression, the monolayers begin to collapse, resulting in modest increases in *Π*. Owing to the hydrophobic nature of the PS NPs, the collapsing PS NP monolayer exhibits successive out‐of‐plane deformations in the order of wrinkling, buckling, and folding as compression proceeds (Figure 1c; and Figure S1b, Supporting Information). This eventually leads to the formation of a folded structure with a size of ≈100 µm (Figure 1c; and Figure S1b, Supporting Information), which is in good agreement with theoretical estimations (for more details, see Discussion S2, Supporting Information). Remarkably, these collapse behaviors of the PS NP monolayers appear unaffected by *P*_de_, because their structures are extruded toward the air, resulting in no entropy loss of depletants (PEG molecules) in the water.

On the other hand, hydrophilic SiO_2_ NPs have a *θ*_wca_ of ≈20° in the absence of PEG in water,^[^ ^39^ ^]^ yielding an extremely small *Π*_max_ of ≈0.26 mN m^−1^ for the SiO_2_ NP monolayer (more details in Discussion S1, Supporting Information). Accordingly, the SiO_2_ NPs are easily desorbed from the air–water (pure) interface under lateral compression, consistent with the result from the compressional Langmuir isotherms (Figure S1a, Supporting Information). In contrast, it has previously been reported that SiO_2_ particles can withstand *Π* to some extent at the air–water interface in the presence of PEG in water^[^ ^29^ ^]^ because PEG molecules in water induce *P*_de_ near the interface, thereby suppressing the desorption of the SiO_2_ particles from the interface.^[^ ^29^, ^33^ ^]^ Additionally, PEG molecules adsorb to the surface of SiO_2_ particles,^[^ ^33^ ^]^ leading to an increase in the *θ*_wca_ of SiO_2_ particles to ≈60°.^[^ ^41^ ^]^ Considering this, *Π*_max_ is derived as *Π*_max_ ∽ (1‐cos*θ*_wca_)^2^·*γ* + *c*·*P*_de_, where *c* represents the characteristic length of the system (more details in Discussion S3, Supporting Information). With estimated values of *P*_de_ ≈ 5 kPa and *c* ≈ 1 µm (Discussion S3, Supporting Information), the calculated *Π*_max_ of the SiO_2_ NPs is ≈25 mN m^−1^. This value is well consistent with the experimental result, although the measured value (≈20 mN m^−1^) from the compressional Langmuir isotherm (Figure 1b) is slightly lower than the calculated one, as in the case of the PS NP monolayer. Additionally, considering that the characteristic length (≈1 µm) corresponds to the length scale of a domain structure where out‐of‐plane deformation occurs (Discussion S3, Supporting Information), the structure formed underneath the residual NP monolayer at the interface, as a result of buckling, should be another single SiO_2_ NP.^[^ ^29^ ^]^ The NPs residing underneath the residual NP monolayer are attractive to each other and also to the monolayer due to *P*_de_. Consequently, a closely packed NP bilayer is formed, as shown in Figure 1d, and further compression on this NP bilayer can also lead to the formation of thicker multilayers.^[^ ^29^ ^]^

For the PS/SiO_2_ NP mixture monolayers, based on the markedly different *Π*_max_ and collapse behaviors of the PS NP and SiO_2_ NP monolayers in the presence of *P*_de_, we postulate that the PS NPs (*Π*_max_ ≈ 50 mN m^−1^) remain at the interface whereas the SiO_2_ NPs (*Π*_max_ ≈ 20 mN m^−1^) are readily desorbed from the interface and subsequently held underneath the residual PS NP layer at the interface, during lateral compression at *Π* in the range of 20–50 mN m^−1^. These differing responses to lateral compression will eventually lead to the formation of a stratified film consisting of a PS NP monolayer atop a SiO_2_ NP multilayer.

### 2.2 Collapse Behaviors of the PS/SiO_2_ NP Mixture Monolayers–Active Stratification

**Figure 2:**
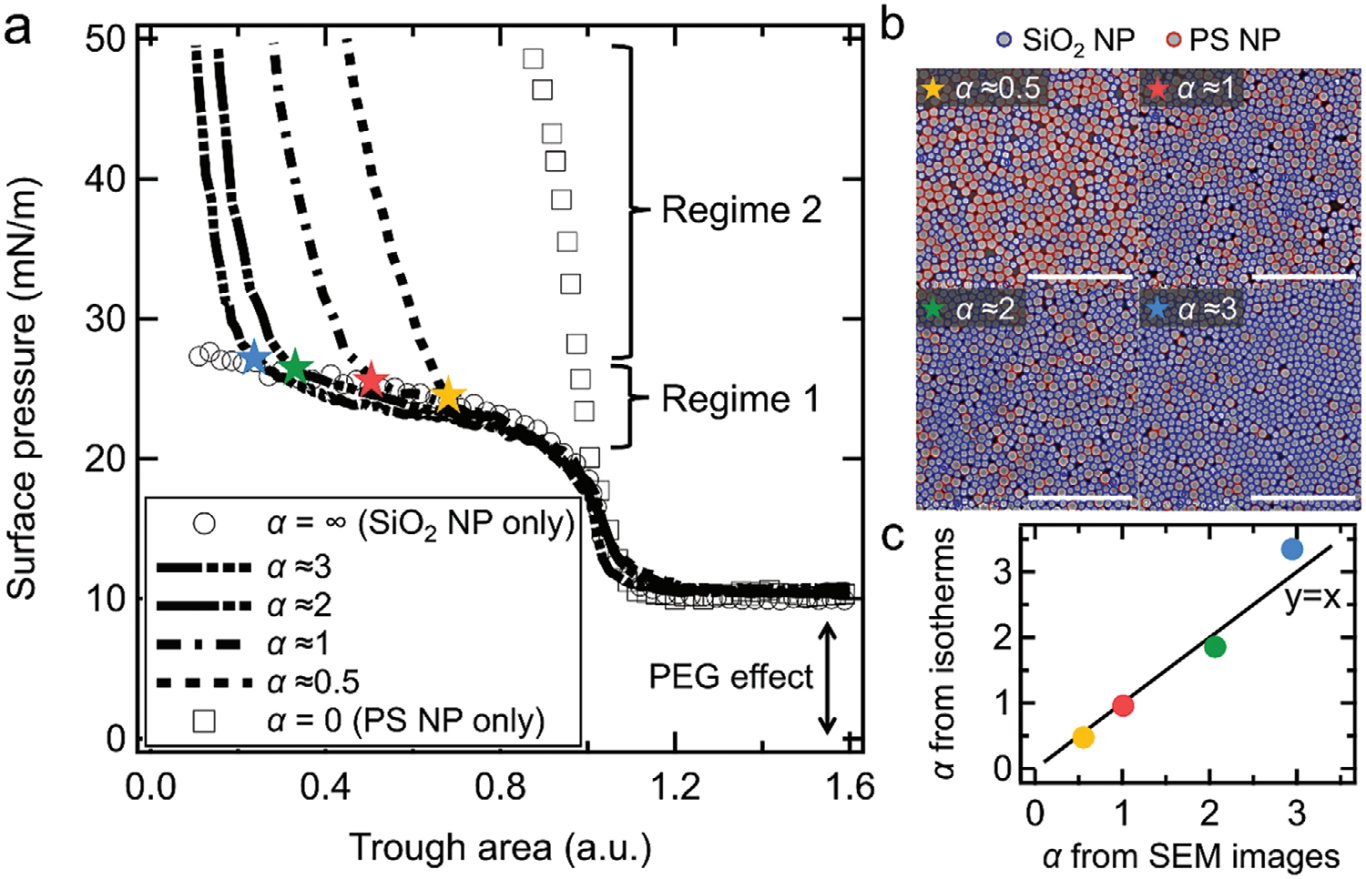
Compressional Langmuir isotherm analysis. a) Compressional Langmuir isotherms of the NP mixtures [PS (diameter = 960 nm) and SiO_2_ (diameter = 700 nm)] at the air–water (containing 0.8 wt% PEG) interface according to the various values of *α*. The regime 1 represents where *Π* increases gently after reaching ≈20 mN m^−1^, and the regime 2 represents where *Π* rises sharply again from *Π* ≈ 25 mN m^−1^. The star markers indicate the point at which the regime 1 ends (i.e., the regime 2 starts). b) SEM images of the well‐packed NP mixture monolayers at the interface with distinction of each type of NP. Each type of NP is distinguished according to the size (PS NP: red circle, SiO_2_ NP: blue circle) using MATLAB with a customized code, and counted to obtain the values of *α* (Figure S2, Supporting Information). All scale bars are 10 µm. c) Correlation between the values of *α* obtained from the isotherms and SEM images. The values of *α* from the isotherms are extracted based on the fact that the regime 2 starts at the trough area (marked by the stars markers) corresponding to the fraction of the area occupied by the PS NPs, 1/(1+*α*).

To examine the collapse behaviors of the PS/SiO_2_ NP mixture monolayers, we analyze the compressional Langmuir isotherms. We prepared the NP mixture monolayers by introducing suspensions of the NP mixtures with varying mixing ratios onto the interface. We define the ratio of the area occupied by the SiO_2_ NPs to that occupied by the PS NPs as *α*, and compare isotherms at various values of *α*, as presented in **Figure** **2**a. For effective comparison, the trough area (*x*‐axis) is normalized relative to the point where the monolayer begins to collapse (*Π* ≈ 20 mN m^−1^). The isotherms remain nearly identical until the NP mixtures reach a close‐packed state, indicated by a sharp increase of *Π* up to ≈20 mN m^−1^, after which they change significantly. In contrast to the isotherms of the pure components, the NP mixtures exhibit two distinct regimes in the isotherms: 1) *Π* increases gently after reaching ≈20 mN m^−1^; 2) *Π* sharply rises again from *Π* ≈ 25 mN m^−1^. The regime 1 aligns well with the isotherm of the collapsing SiO_2_ NP monolayer, which presumably implies that only the SiO_2_ NPs collapse out of the NP mixture monolayers. On the other hands, the regime 2 likely arises from the close‐packing of the PS NPs that can withstand *Π* up to ≈50 mN m^−1^. This can also be supported by the fact that the regime 2 begins at the trough area (marked by the stars of different colors) corresponding to the fraction of the area occupied by the PS NPs, 1/(1+*α*). In addition, the values of *α* extracted from the isotherms in this manner closely match the actual *α* values obtained by direct counting (Figure 2b; and Figure S2, Supporting Information), as shown in Figure 2c. Based on the understanding from the isotherm analysis, we predict that a fully stratified film comprised with a PS NP monolayer atop SiO_2_ NP layers will be achieved when the NP mixture monolayers are compressed to the end of regime 1.

**Figure 3:**
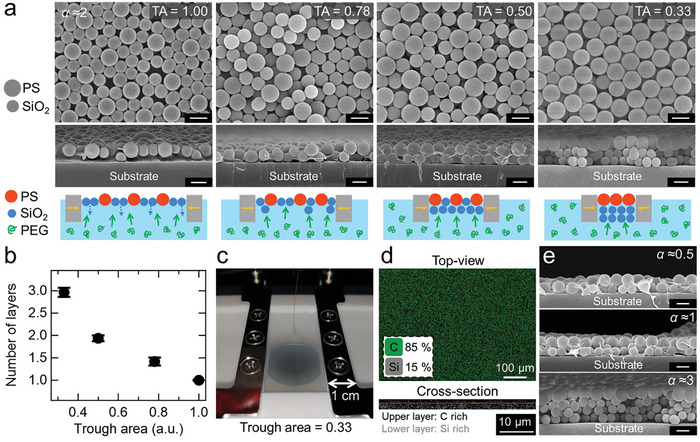
Structural characterization of the NP mixture [PS (diameter = 960 nm) and SiO_2_ (diameter = 700 nm) NPs] layers at the air–water (containing 0.8 wt% PEG) interface. a) SEM images with schematic illustrations as a function of TA at *α* ≈ 2. As the TA is reduced, the number of the SiO_2_ NPs underneath the PS NPs increases, and then stratification into a PS NP monolayer atop a double layer of the SiO_2_ NPs is achieved at the end of regime 1 (TA = 0.33). b) The number of layers extracted from the surface profiles (Figure S3, Supporting Information) as a function of TA at *α* ≈ 2. The error bars represent the standard deviation of the number of layers obtained from 10 different profiles. c) A photographic image of the NP mixture layer (*α* ≈ 2) at the interface when the TA is 0.33. d) Top‐view and cross‐sectional element‐mapped SEM images of the NP mixture films at a TA of 0.33. e) Cross‐sectional SEM images of the stratified NP films with various values of *α*, deposited at the end of regime 1 (Figure 2a). The stratified NP films are composed of a PS NP monolayer atop *α* layers of the SiO_2_ NPs. All scale bars are 1 µm unless otherwise noted.

The actual structures of the NP mixture layers at the interface according to compression are visualized using a SEM after transferring the layers onto solid substrates. The SEM images in **Figure** **3**a show notable changes in the NP mixture monolayer (*α* ≈ 2) as it is compressed to a trough area (TA) of 0.33, marking the end of the regime 1. When the close‐packed NP mixture monolayer at a unit TA is compressed to a TA of 0.78, a few SiO_2_ NPs underneath the PS NPs are first observed. With further compression to a TA of 0.50, the number of such SiO_2_ NPs underneath the PS NPs increases significantly, and ultimately a bilayer of the SiO_2_ NPs is predominantly observed underneath a PS NP monolayer when the TA reaches 0.33. These observations strongly support the abovementioned argument based on the isotherm results. We therefore firmly believe that only the SiO_2_ NPs are actively pushed downward into the water from the interface as a result of the buckling of the SiO_2_ NP domains in the NP mixture monolayers yet are held underneath the residual NP monolayer at the interface owing to *P*_de_, as schematically illustrated in Figure 3a. This process continues until there are no remaining SiO_2_ NPs in the residual NP monolayer, eventually leading to a complete stratification of the NPs into a PS NP monolayer atop the SiO_2_ NP layers. Additionally, based on this intriguing stratification mechanism, the number of NP layers during compression to the end of regime 1 can be simply estimated as 1/trough area, which agrees well with the results from the SEM images (Figure 3a) and the surface profiles of the NP films (Figure 3b; and Figure S3, Supporting Information).

When the NP mixture monolayer with *α* ≈ 2 is fully stratified at a TA of 0.33, the NP mixture layer at the interface appears flat and homogeneous to the naked eye, as shown in Figure 3c, whereas at TAs between 1.00 and 0.50, the NP mixture layers do not appear flat due to the nonuniform thickness of the layers (Figure S4, Supporting Information). This possibly suggests that the stratification occurs uniformly over the whole interfacial area. Large‐scale stratification is further evident in the lower‐magnification SEM images (Figure S5, Supporting Information), as well as the element‐mapped top‐view SEM image (Figure 3d) presenting ≈85% detection of carbon, which is the primary constituent of PS NP. Given that hexagonal and random packing of circles correspond to ≈91% and 82%,^[^ ^42^ ^]^ respectively, the X‐rays used for element mapping can still infiltrate into the interstices of the PS NPs. This allows for the detection of some silicon constituting the underlying SiO_2_ NP, even though the PS NPs are located extremely densely in the top layer. The element‐mapped cross‐sectional image displayed in Figure 3d also supports large‐scale stratification. It should be noted that the scalability of stratified NP film fabrication using the active stratification process depends entirely on the dimension of the Langmuir trough used,^[^ ^35^ ^]^ and accordingly, better scalability can be achieved by simply employing a larger Langmuir trough.

### 2.3 Controllability of the Active Stratification Process

Given the definition of *α*, the stratified NP film consists of a PS NP monolayer atop *α* layers of the SiO_2_ NPs at the end of regime 1 (indicated by star markers in Figure 2a), which strongly suggests that the number of layers can be readily controlled by adjusting the value of *α*. For *α* ≈ 0.5, the area of the SiO_2_ NPs is half that of the PS NPs, allowing about half of the PS NPs to rest atop the SiO_2_ NPs, as presented in Figure 3e (top). As *α* increases to 1 and 3, stratified films are obtained with PS NP monolayers atop a monolayer and a tri‐layer of the SiO_2_ NPs, respectively (Figure 3e). These observations demonstrate that the number of SiO_2_ NP layers residing underneath the PS NP monolayer can be easily controlled as long as a Langmuir trough can compress to the end of regime 1.

On the other hand, regarding the PEG concentration, which could be another crucial parameter controlling the structure of the stratified films, it should be noted that despite significant variations in the PEG concentration, the structures of the stratified NP films remain highly consistent. When the PEG concentration decreases by 80‐fold from 0.8 to 10^−2^ wt%, *P*_de_ also decreases by 80‐fold (Discussion S3, Supporting Information), while the *E*_comp_ of the SiO_2_ NPs remains almost unchanged (Figure S6a, Supporting Information). Consequently, the length scale of the SiO_2_ NP domain in which out‐of‐plane deformation occurs (≈characteristic length, *c*) increases from ≈1 to ≈3 µm (for more details, see Discussion S3, Supporting Information). However, the thickness of the stratified NP films remains unaffected, especially for relatively low values of *α*, as shown in Figure S6b (Supporting Information). This is likely because the SiO_2_ NPs form small domains composed of only a few in the mixture monolayers (Figure 2b) so that the structures underneath the residual NP monolayers at the interface do not obey the scaling physics derived from the classic theory for a thin elastic film floating at the interface.^[^ ^29^, ^43^ ^]^ This reasoning is supported by the occasional observation of considerably thicker structures than the surroundings in the stratified NP films with *α* ≈ 3 (Figure S6b, Supporting Information), where considerably large SiO_2_ NP domains exist. Furthermore, our experimental setup allows successful stratification down to a PEG concentration of 10^−3^ wt%, as presented in Figure S7 (Supporting Information). At a PEG concentration of 10^−4^ wt%, *P*_de_ does not appear to effectively suppress the desorption of the SiO_2_ NPs from the interface, possibly due to the vibrations generated during the compression of the Langmuir trough.

### 2.4 Universality of the Active Stratification Process

**Figure 4:**
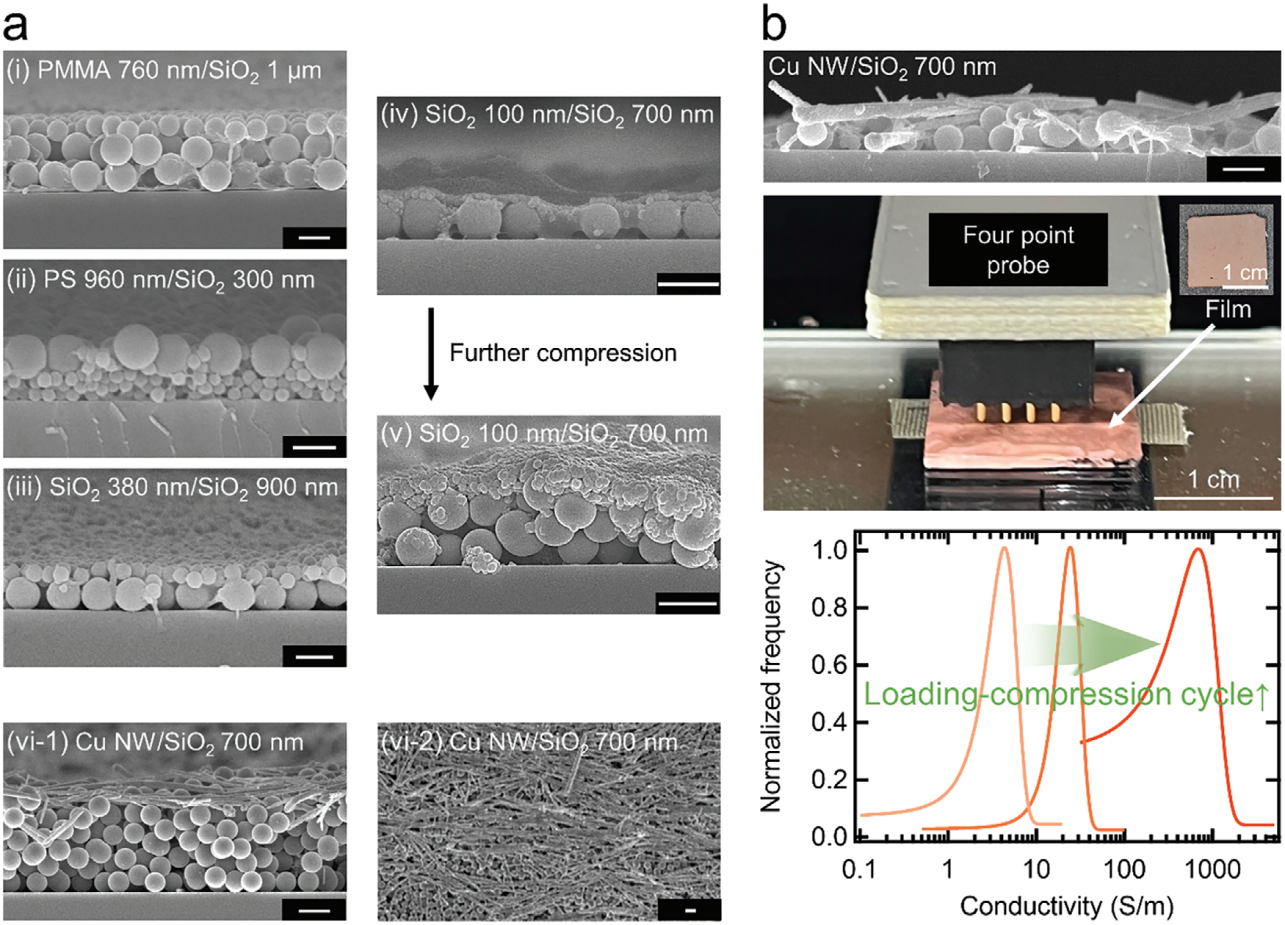
Universality of the active stratification process. a) SEM images of the stratified NP films of various NP combinations manufactured at the air–water (containing 0.8 wt% PEG) interface by the active stratification process. The compositions of the stratified films are described by the following nomenclature: type and diameter of more interfacial active NP/type and diameter of less interfacial active NP. The Cu NW has a diameter of 100 nm and a length of 10–20 µm. All scale bars are 1 µm unless otherwise noted. In (a), the surfaces of the SiO_2_ NPs with diameters of 380 and ≈100 nm are treated with dichlorodimethylsilane to increase interfacial activity. b) Electrical conductivity of the stratified films composed of Cu NW monolayers atop SiO_2_ NP monolayers depending on the material loading‐compression cycle. The top panel presents a representative cross‐sectional SEM image of the stratified film obtained with one loading‐compression cycle. The middle panel shows a photographic image of the electrical conductivity measurement of the stratified film. 200 data points were measured, and their frequencies are shown in the form of a Gaussian function (bottom panel).

In our active stratification process, the key condition to be considered for NPs is the difference in interfacial activity quantified by *Π*_max_. As long as the difference in *Π*_max_ between each pure NP monolayer exists and is greater than potential change in *Π* that occurs during the collapse state of the less interfacial active NPs, active stratification can work effectively. This allows for substituting the pair of 960 nm‐PS NPs and 700 nm‐SiO_2_ NPs with other pairs of NPs demonstrating different *Π*_max_ values (Figure S8, Supporting Information). For instance, a substituted pair of smaller polymeric NPs [polymethyl methacrylate (PMMA) NPs] and larger SiO_2_ NPs, compared to the original NP pair, is successfully stratified, as presented in **Figure** **4**a(i). Of course, a pair of NPs with large size differences (>3 times), primarily utilized in the self‐stratification process exploiting a drying binary colloidal mixture, can also be stratified [Figure 4a(ii)]. For a pair of NPs of the same type, the active stratification process can be applied by tuning the interfacial activity of one NP through surface treatment. For example, a pair of SiO_2_ NPs, one untreated and the other treated with dichlorodimethylsilane, which imparts interfacial activity with *θ*_wca_ of ≈90°,^[^ ^44^ ^]^ is stratified as shown in Figure 4a(iii).

Serendipitously, we find that if the surface of ≈100 nm sized SiO_2_ NPs is treated with an increased amount of dichlorodimethylsilane (Experimental Section), their monolayer at the air–water interface becomes thicker upon lateral compression, leading to the formation of a NP multilayer (Figure S9, Supporting Information). This phenomenon is likely because the NPs are desorbed toward the air, possibly owing to the low adsorption energy at the interface, as indicated by the increased compressibility in the compressional Langmuir isotherm (Figure S9a, Supporting Information). When utilizing these NPs alongside untreated SiO_2_ NPs in a pair for the active stratification process, a stratified film is mainly formed with the upper NP layer as a monolayer, consistent with other NP pairs, as depicted in Figure 4a(iv). Intriguingly, with further compression, this film evolves into a stratified film where both the upper and lower NP layers are multilayers, as presented in Figure 4a(v). This strongly suggests that the untreated SiO_2_ NPs are preferentially desorbed from the interface first, forming a typical stratified film with the upper NP layer as a monolayer, but subsequently, both treated and untreated NPs become thicker together, as compression proceeds. Nevertheless, we believe that more systematic analysis and understanding are needed to determine a more precise mechanism for this phenomenon.

Moreover, shape‐anisotropic nanowires (NWs) can also be employed for the active stratification, as displayed in Figure 4a(vi). Given the challenges in estimating and calculating the diffusivity and interactions of shape‐anisotropic colloids^[^ ^45^ ^]^ for application in the self‐stratification process exploiting a drying binary colloidal mixture, it is a significant advantage that there are no restrictions on particle shape. The stratified film displayed in Figure 4a(vi) features a top monolayer of interconnected interfacial active Cu NWs, suggesting that the film is electrically conductive. The degree of interconnectivity, which determines the electrical conductivity, is likely to increase with the packing density of NWs.^[^ ^46^ ^]^ Hence, we attempt to increase the packing density by repeating material loading‐compression cycles^[^ ^47^ ^]^ in manufacturing the stratified films composed of Cu NW monolayers atop SiO_2_ NP monolayers shown in Figure 4b. As anticipated, the conductivity increases with each loading‐compression cycle, reaching ≈1000 S m^−1^ after 3 cycles (Figure 4b), which is comparable to the conductivities reported for other aligned metallic NW monolayers.^[^ ^48^, ^49^ ^]^ Considering both the conductivity value and the thickness advantage of the NW monolayer, the stratified film could find application as transparent electrodes, particularly in touch screens where extremely high conductivity is not essential.^[^ ^50^ ^]^

### 2.5 Interfacial Annealing of the Stratified NP Layers

**Figure 5:**
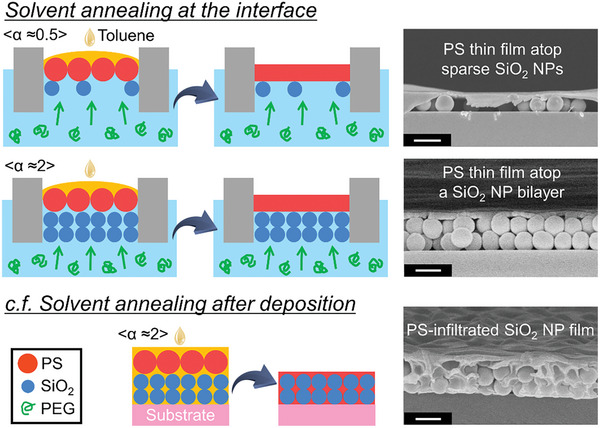
Schematic illustrations and cross‐sectional SEM images of the stratified NP mixture [PS (diameter = 960 nm) and SiO_2_ (diameter = 700 nm) NPs] layers at the air–water (containing 0.8 wt% PEG) interface upon the interfacial solvent annealing. Toluene floats on the air–water interface and anneals the PS NPs only at/above the interface, resulting in a clearly stratified film composed of a thin PS film atop the sparse SiO_2_ NPs or SiO_2_ NP bilayers at *α* ≈ 0.5 or ≈2, respectively. In contrast, the stratified NP layer is annealed after deposition, which leads to a PS‐infiltrated SiO_2_ NP film. All scale bars are 1 µm.

An additional feature of the active stratification process is the composition of the stratified NP layers, with one NP layer at the interface and the other below the interface. This unique orientation allows for selective solvent annealing of one NP layer without influencing the other NP layer. In the case of stratified layers of the PS NPs and SiO_2_ NPs, only the PS NPs situated at the interface can be annealed by introducing a solvent for PS, which is lighter than water, onto the interface (**Figure** **5**). Although the PS NPs become fluidized, the fluidized PS cannot infiltrate into the interstices of the SiO_2_ NPs located below the interface due to its immiscibility with water. As a result, clearly stratified layers composed of thin PS layers atop the sparse SiO_2_ NPs and the SiO_2_ NP bilayer are successfully achieved for *α* ≈ 0.5 and 2, respectively. Notably, when using a small amount of solvent that partially wets the air–water interface, we can observe annealed and nonannealed regions corresponding to wetting and nonwetting regions (Figure S10, Supporting Information), suggesting the potential of patterning.

Such stratified layers have not been previously realized through sequential coating processes utilizing conventional coating methods such as spin coating, dip coating, spray coating, or flow coating etc., because polymer solutions/melts can infiltrate into the interstices of NPs through capillarity or gravity.^[^ ^51^ ^]^ Even post‐solvent/thermal annealing on a stratified NP film after deposition also results in capillary rise infiltration of the fluidized PS into the interstices of the SiO_2_ NPs,^[^ ^52^ ^]^ as presented in Figure 5. Furthermore, through Langmuir–Blodgett (down‐stroke)^[^ ^53^ ^]^ or Langmuir–Schaefer^[^ ^54^ ^]^ techniques, the stratified layer consisting of a polymer thin layer on top of a NP layer at the interface can be transferred in a reversed order. We believe that these thin films hold significant potential for transparent and flexible electrodes using conductive particles/wires/flakes.^[^ ^55^, ^56^ ^]^

## 3 Conclusion

In this study, we have introduced an active stratification process that stratifies colloidal NPs with different interfacial activity by leveraging the air–water interface where *P*_de_ comes into play. As the NP monolayer formed at the interface undergoes collapse by lateral compression, only the less interfacial active NPs are actively pushed downward into the water yet held underneath the residual monolayer at the interface thanks to *P*_de_. This segregation continues until the interface becomes free of the NPs with less interfacial activity, eventually leading to a fully stratified film comprised of a layer of more interfacial active NPs atop one or more layers of less interfacial active NPs. The active stratification process is not only universal and scalable, but also controllable depending on the area fraction of each NP and the degree of compression. Unlike conventional stratification processes that exploit a sequential deposition of each layer or a drying binary colloidal mixture, the active stratification process does not require any time‐consuming iterative processes or intricate control of operating conditions. Furthermore, the numerous available NP pairs and depletants greatly expand the applicability of asymmetric NP multilayers.

By replacing PEG with biocompatible hydrophilic polymers, such as dextran and polyvinyl alcohol, stratified asymmetric NP multilayers are expected to be applicable in biocompatible and eco‐friendly systems. In particular, for drug encapsulation and controlled release applications, multilayer coatings that prevent mixing between layer components are highly desirable, so it is expected that the active stratification process enables the preparation of coatings containing active components in the top layer and inactive components in the bottom layer, possibly facilitating the manufacture of effective drug encapsulations. In addition, when combined with a solvent annealing process, this stratification process can allow for the creation of films consisting of a polymer layer on top of a conductive particle layer, which can be applied to transparent and flexible electrodes. By introducing biomolecules and quantum dots as depletants, we envision that active stratification could add extra functionalities to the electrodes, facilitating biosensing and display applications, respectively.^[^ ^57^, ^58^, ^59^ ^]^

## 4 Experimental Section

### Materials

Aqueous dispersions of the PS NP (PS 0 4001, diameter = 960 nm, 10% w/v) and the PMMA NP (PMMA‐R‐0.75, diameter = 760 nm, 5 wt %) were obtained from Bangs Laboratories (Fishers, IN) and microParticles (Berlin, Germany), respectively. 10% w/v aqueous dispersions of the SiO_2_ NPs with diameters of 300 nm (24 321‐15), 700 nm (24 324‐15), 900 nm (24 325‐15), and 1000 nm (24 326‐15) were all procured from Polysciences (Warrington, PA). An ethanol dispersion of the Cu NW (diameter = 100 nm, length = 10–20 µm, 5 mg mL^−1^), PEG (81 310, average *M*_n_ = 35 000 g mol^−1^, polydispersity = 1.26), ethanol (ACS reagent, ≥99.5%), toluene (anhydrous, 99.8%), and dichlorodimethylsilane (≥99.5%) were purchased from MilliporeSigma (St. Louis, MO). The silanized SiO_2_ NPs were prepared as follows: 0.2 g of SiO_2_ NPs, synthesized using the Stöber method,^[^ ^60^ ^]^ were dispersed in 10 mL of toluene. For silanization, 0.000 857–0.05 wt% of dichlorodimethylsilane was added to the dispersion, which was then stirred at 300 rpm overnight. After recovering the silanized SiO_2_ NPs by centrifugation, the NPs were washed 5 times with ethanol and dried at 60 °C.

### Spreading Suspension Preparation

All the NP dispersions were rinsed prior to use according to the following procedure to remove possible residual chemicals: 1) The NP dispersion was centrifuged at 10 000 rpm for 3 min; 2) the supernatant solution was replaced by the same amount of ethanol; 3) the precipitated NPs were redispersed in ethanol using a vortex machine (VM30, DAIHAN Scientific, Wonju, Republic of Korea); 4) Steps 1–3 were repeated four more times. Since the synthesized silanized SiO_2_ NP were obtained in a powder form, the procedure started after dispersing them in ethanol at 10% w/v. The rinsed NP ethanol dispersions were mixed with each other at a concentration of ≈10% w/v in the desired NP ratio.

### Langmuir Isotherm Measurement and Stratification of NP Monolayers

A customized Langmuir trough with an adjustable interfacial area between 4 and 48 cm^2^ was filled with a PEG aqueous solution at a desired concentration (0–0.8 wt%), and then 20 µL of the prepared spreading suspension was gently introduced onto the air–water interface using a microliter syringe (7637‐01, Hamilton, Reno, NV) combined with a stainless‐steel needle (7758‐01, Hamilton). After waiting for 15 min for the ethanol to evaporate and for the NP mixture monolayer to stabilize, the monolayer was laterally compressed at a rate of ≈3 cm^2^ min^−1^ to the desired trough area by two moving barriers. In the meantime, surface pressure was measured using a Wilhelmy plate tensiometer (Riegler & Kirstein, Potsdam, Germany). The correlation between the trough area and surface pressure was monitored using a customized code in LabVIEW to draw a Langmuir isotherm. When repeating the material loading‐compression cycle, 5 µL of the prepared spreading suspension was used, and the rest of the procedure was kept constant. For the annealing of the PS NPs in the compressed NP mixture layer, 10 µL of toluene was gently introduced onto the interface where the NP mixture layer was present using the microliter syringe.

### NP Layer Deposition from the Air–Water Interface to Substrates

The NP mixture layers at the air−water interface were deposited to mica substrates (highest grade mica disk, 20 mm, Ted Pella, Redding, CA) by the popular inverted Langmuir–Schaefer technique.^[^ ^35^ ^]^ A mica substrate attached on a glass supporting fixture (diameter = 20 mm, height = 10 mm) was submerged in the PEG solution before forming the initial NP monolayer. When the initial NP monolayer was compressed to the desired trough area, the PEG solution outside the barriers was gently sucked out using an aspirator. As the height of the interface became lower than that of the mica substrate, the NP layer at the interface was successfully transferred to the substrate. The mica substrate was sometimes replaced with a silicon wafer (WSI0PR0029, iTASCO, Seoul, Republic of Korea) cleaved into ≈20 × 20 mm^2^ for the purpose of cross‐sectional observation, because a silicon wafer is readily segmented.

### NP Layer Characterization

The macrostructures of the NP layers during compression at the air–water interface were visualized using a customized microscope equipped with a CCD camera (WAT‐902H, Watec, Tsuruoka, Japan). The microstructures of the NP layers were analyzed using a field emission scanning electron microscope (FE‐SEM, S‐4800, Hitachi, Tokyo, Japan) at 5 kV electron beam voltage after deposition. To obtain high resolution images, all the samples were coated with 10 nm of platinum using a sputter coater (SPT‐20, COXEM, Daejeon, South Korea), which improves the electron conductivity. The chemical elements that compose the NP layers were identified by energy dispersive X‐ray spectroscopy (EDS) analysis using an EDS detector (Octane Elite, AMETEK, Berwyn, PA) coupled with a FE‐SEM (SU8230, Hitachi). In the EDS analysis, the NP layers were deposited on gold substrates (16 208‐G, Ted Pella) and the cleaved silicon wafers for top‐down and cross‐sectional observations, respectively. After 10 nm of osmium coating using an osmium coater (HPC‐1SW, Vacuum Device, Mito, Japan), EDS analysis was performed at 5 kV electron beam voltage that limited the beam penetration depth to be smaller than the sizes of the NPs. The surface profiles of the NP layers were obtained using a stylus profilometer (Dektak 8, Veeco, Plainview, NY) with a resolution range of 0.083–0.278 µm depending on the scan length. The electrical conductivity of the stratified films composed of Cu NWs was measured using a four‐point probe system (T2001A4, Ossila, Sheffield, UK).

## Conflict of Interest

The authors declare no conflict of interest.

## Supporting information

Supporting Information

## Data Availability

The data that support the findings of this study are available from the corresponding author upon reasonable request.
