# Supplementary material for: Active Stratification of Colloidal Mixtures for Asymmetric Multilayers
Source: Small. 2024 Aug 16;20(52):2404348. doi: 10.1002/smll.202404348 (PMC11673404; doi:10.1002/smll.202404348)
Supplement: Supplementary file 1 — Supporting Information [file SMLL-20-2404348-s001.docx]

Supporting Information

**Active Stratification of Colloidal Mixtures for Asymmetric Multilayers**

Baekmin Q. Kim, Jongmin Q. Kim, Hojoon Yoon, EunSuk Lee, Siyoung Q. Choi,* and KyuHan Kim*

**Table of Contents**

Discussion S1–S3

Figure S1–S10

SI references

**Discussion S1. Maximum surface pressure (*Π*_max_) of the nanoparticles (NPs) adsorbed at the air**-**water interface.**

*Case 1. In the absence of polyethylene glycol (PEG)* *in the water*

*Π*_max_ of the NPs adsorbed at the air-water interface is derived as *Π*_max_ ~ (1-cos*θ*_wca_)^2^·*γ* (*θ*_wca_: water contact angle, *γ*: air-water interfacial tension) from the balance between the adsorption energy [*E*_ad_ ~ *c*^2^·(1-cos*θ*_wca_)^2^·*γ*]^[1]^ and the energy applied by compression (~ *c*^2^·*Π*), where *c* is the characteristic length of the system. Considering the *θ*_wca_ of the PS and SiO_2_ NPs are ≈90 ° and ≈20 °,^[2]^ respectively, *Π*_max_ of the PS NPs is ~ *γ*, which means that the PS NPs can withstand *Π* up to *Π*_max_ ≈72 mN/m, whereas *Π*_max_ of SiO_2_ NPs is ~ 0.0036*γ*, which means that the SiO_2_ NPs can hardly withstand *Π* (*Π*_max_ ≈0.26 mN/m) and are readily desorbed from the interface (Figure S1a).

*Case 2. In the presence of PEG in the water*

When PEG molecules are present in the water, they function as a depletant, exerting depletion pressure (*P*_de_) on the NPs adsorbed at the air-water interface.^[3]^ For the PS NPs, as mentioned in the main text, they are not affected by *P*_de_, because their structures are extruded towards the air. Accordingly, the same *Π*_max_ estimation as in case 1 can be applied, and given the almost unchanged *θ*_wca_,^[4]^ *Π*_max_ ~ *γ* ≈62 mN/m. In contrast, the SiO_2_ NPs are affected by *P*_de_, which will be discussed in detail in Discussion S3.

**
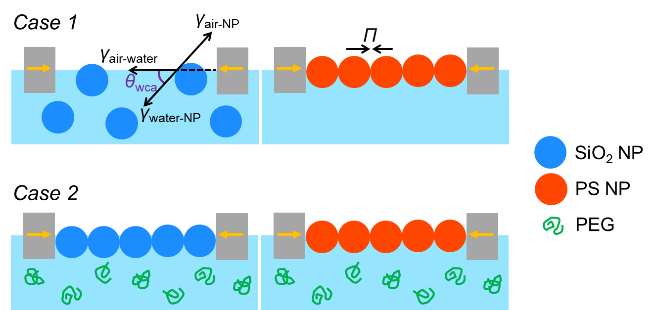
**

**Discussion S2. Theoretical estimation of the specific wavelength of the collapsing PS NP monolayers at the air**-**water interface.**

When a thin elastic film floating at the air-water interface is compressed, wrinkles with a specific wavelength [*λ* ~ (*B*/*K*)^1/4^, *B*: bending stiffness of the film, *K*: subphase stiffness (*K* = *ρ*·*g*, *ρ* is water density, *g* is gravitational acceleration)] emerge as a linear out-of-plane deformation.^[5]^ For a colloidal particle monolayer, *B* is possibly scaled as *E*_comp_·*a*^2^, where *E*_comp_ is the compressional modulus of the film, and *a* is the particle radius.^[3]^ In our system, when the PEG molecules present in the water, the *E*_comp_ of the PS NP monolayer is 444 mN/m from the compressional Langmuir isotherm (Figure 1b), which yields *λ* ≈100 μm that is consistent with the observed values (Figure 1d). Since the wavelength indicates the length of a unit domain in which out-of-plane deformation occurs, it can be regarded as the characteristic length of the system (*c*). It is also known that the transition from wrinkling to non-linear out-of-plane deformations of buckling and folding occurs by further compression, and the vertical size of the fold is on a scale similar to the wavelength^[5]^. The observed fold size is ≈100 μm (Figure 1d), which is in good agreement with the theoretical estimation. Even when the water does not contain the PEG molecules, the *E*_comp_ is similar as 340 mN/m (Figure S1a), yielding *λ* ≈100 μm that is consistent with the observed values (Figure S1b).


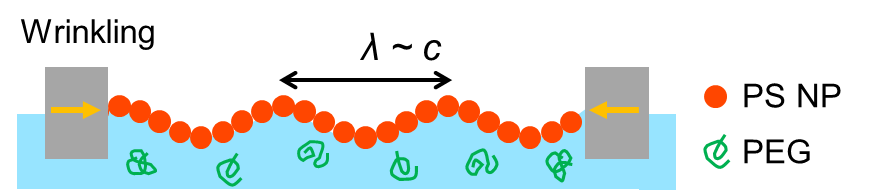


**Discussion S3. *Π*_max_ and characteristic length of the SiO_2_ NPs adsorbed at the air**-**PEG solution interface.**

In this work, the PEG molecules (MW = 35,000 g·mol^-1^) function as a depletant, and cause *P*_de_ near the interface: *P*_de_ suppresses the desorption of the SiO_2_ NPs from the interface, which causes entropic loss of the surrounding PEG molecules.^[3]^ The magnitude of *P*_de_ is ideally scaled as osmotic pressure, *n*·*R*·*T* (*n*: number concentration of depletants, *R*: gas constant, *T*: temperature), however, in practice it is about 10 times greater when the excluded volume is taken into account.^[6–8]^ To maximize *P*_de_ so as to suppress the desorption of the NPs with as large a pressure as possible, the PEG concentration is set to 0.8 wt %, given that the NPs poorly adsorb to the interface at a concentration above that due to too many PEG molecules adsorbed at the interface.^[3]^ At the PEG concentration of 0.8 wt %, the magnitude of *P*_de_ is ≈5 kPa in practice.

Considering the energy that *P*_de_ suppresses the desorption of the SiO_2_ NPs per unit domain, *c*^3^·*P*_de_, the *Π*_max_ derived in Discussion S1 is newly given as *Π*_max_ ~ (1-cos*θ*_wca_)^2^·*γ* + *c*·*P*_de_. *c* was estimated as *c* ~ *λ* ~ (*E*_comp_·*a*^2^/*K*)^1/4^ in Discussion S2 for the PS NPs, but in contrast, *P*_de_/*a* possibly works as the effective *K* for the SiO_2_ NPs,^[3]^ leading to *c* ~ *λ* ~ [*E*_comp_·*a*^2^/(*P*_de_/*a*)]^1/4^. The *E*_comp_ of the SiO_2_ NP monolayer is obtained as 180 mN/m from the compressional Langmuir isotherm (Figure 1b), which yields *c* ≈1 μm. The PEG molecules dissolved in the water adsorbs to the surface of the SiO_2_ NPs,^[6]^ and thereby the *θ*_wca_ of the SiO_2_ NP is presumably increased to ≈60 °.^[9]^ Accordingly, the *Π*_max_ is ≈25 mN/m, which agrees well with the experimental result shown in Figure 1b.


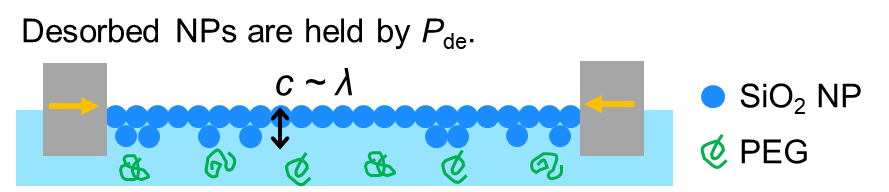


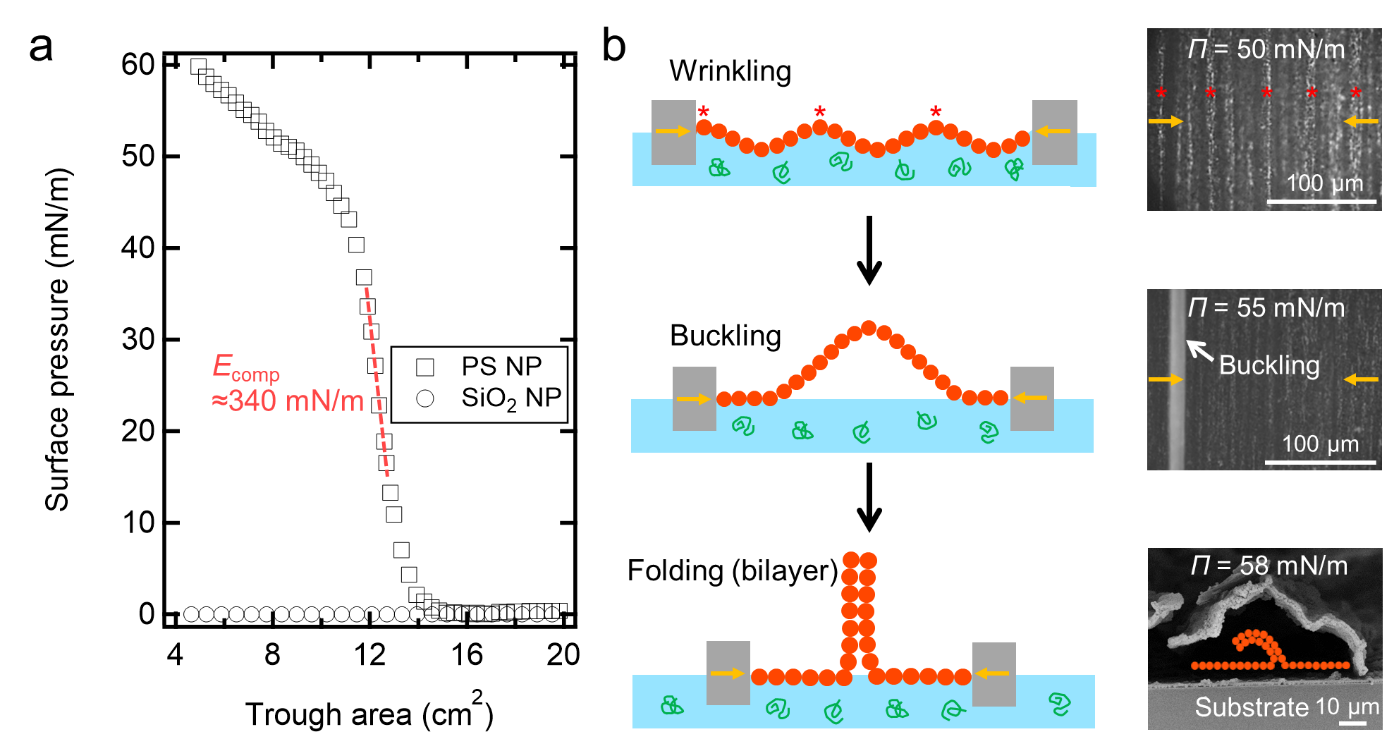


Figure S1. a) Compressional Langmuir isotherm of the PS (diameter = 960 nm) and SiO_2_ (diameter = 700 nm) NPs at the air-water interface. The PS NP monolayer withstands *Π* up to ≈50 mN/m and then collapses by further compression, whereas the SiO_2_ NP monolayer cannot withstand any *Π*. The *E*_comp_ is calculated as *E*_comp_ = -*A*·(d*Π*/d*A*), where *A* is the trough area, when the close-packed monolayer is compressed. b) Schematic illustration and corresponding images of the collapsing PS NP monolayer according to lateral compression at the interface. The collapsing PS NP monolayer exhibits out-of-plane deformations in the order of wrinkling, buckling, and folding as compression proceeds. The images of the wrinkling and buckling are top-down optical microscope images, and the image of the folding is a cross-sectional scanning electron microscope (SEM) image. The asterisk makers indicate the crest points of the wrinkles.

| **Number of the SiO_2_ NPs** | **Number of the PS NPs** | ***α*** |
| --- | --- | --- |
| 444.4±19.7 | 427.4±15.4 | 0.553 |
| 674.7±42.0 | 355.5±18.9 | 1.009 |
| 814.7±42.4 | 210.0±11.2 | 2.063 |
| 908.9±45.6 | 164.1±12.9 | 2.945 |

**
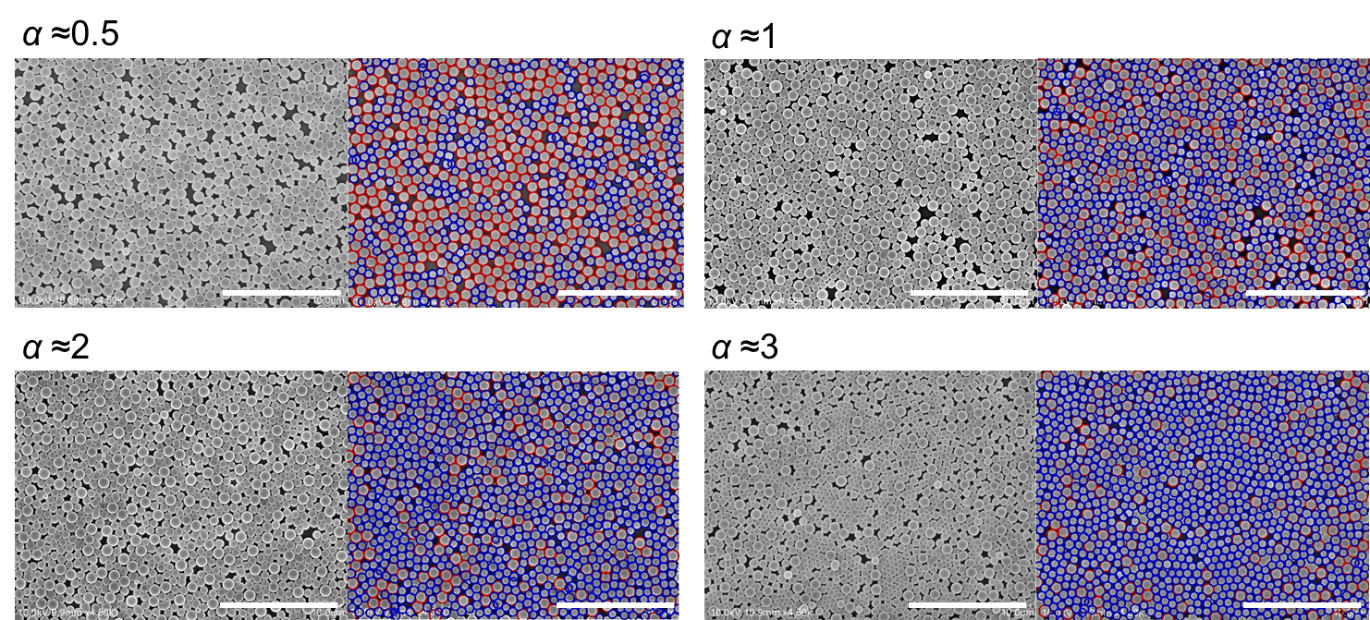
**

**Figure S2.** SEM images of the well-packed NP mixture [PS (diameter = 960 nm) and SiO_2_ (diameter = 700 nm)] monolayers at the air-water (containing 0.8 wt % PEG) interface with distinction of each type of NP, and the values of *α* obtained from the images. The monolayers are deposited at *Π* of ≈15 mN/m (Figure 2a). Each type of NP is distinguished according to the size (PS NP: red circle, SiO_2_ NP: blue circle) using MATLAB with a customized code, and counted to obtain the values of *α*. SEM images of ten different spots are used to statistically yield the values of *α*. All scale bars are 10 μm.


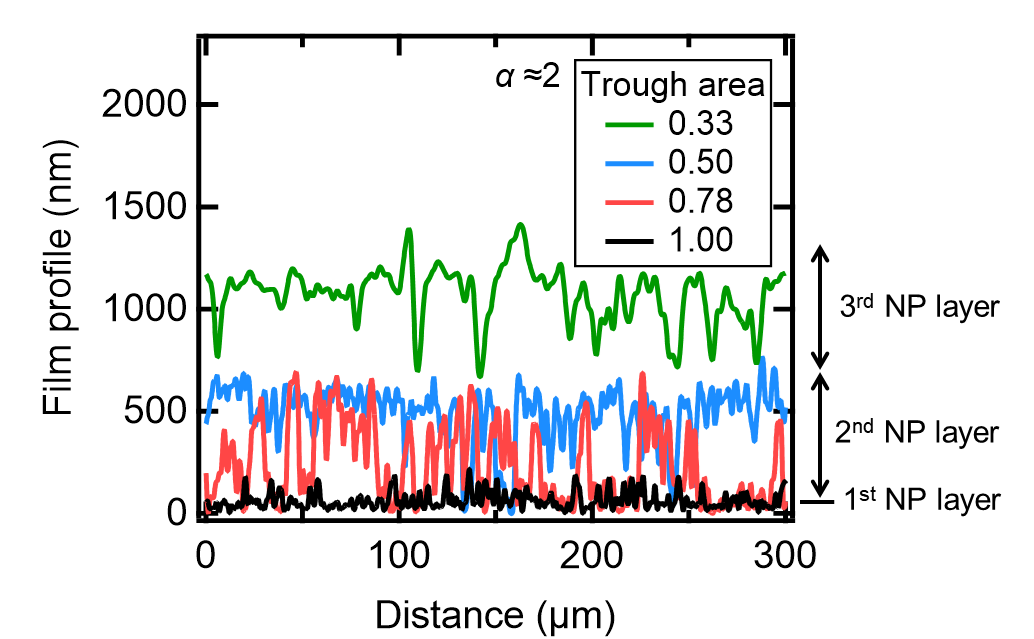


Figure S3. Surface profiles of the NP mixture [PS (diameter = 960 nm) and SiO_2_ (diameter = 700 nm)] layers (*α* ≈2) at the air-water (containing 0.8 wt % PEG) interface according to the trough area. For the trough area of 1.00, 0.78 and 0.50, the surface profiles are measured based on the bottom NP layer. For the trough area of 0.33, the surface profiles are measured based on the 2^nd^ NP layer from the substrate, and shifted to the upward to be positioned right over the surface profiles of the 2^nd^ NP layer.


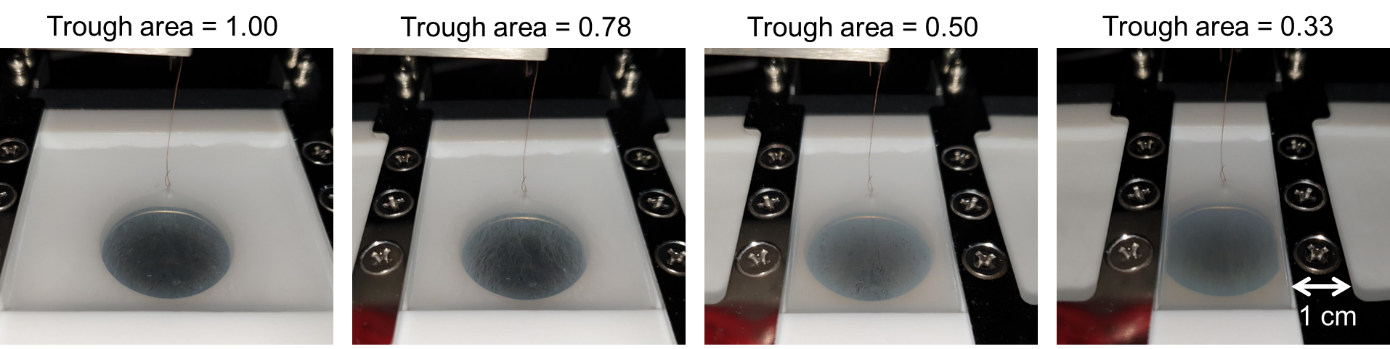
 Figure S4. Photographic images of the NP mixture [PS (diameter = 960 nm) and SiO_2_ (diameter = 700 nm)] layers (*α* ≈2) at the air-water (containing 0.8 wt % PEG) interface according to the trough area.

**
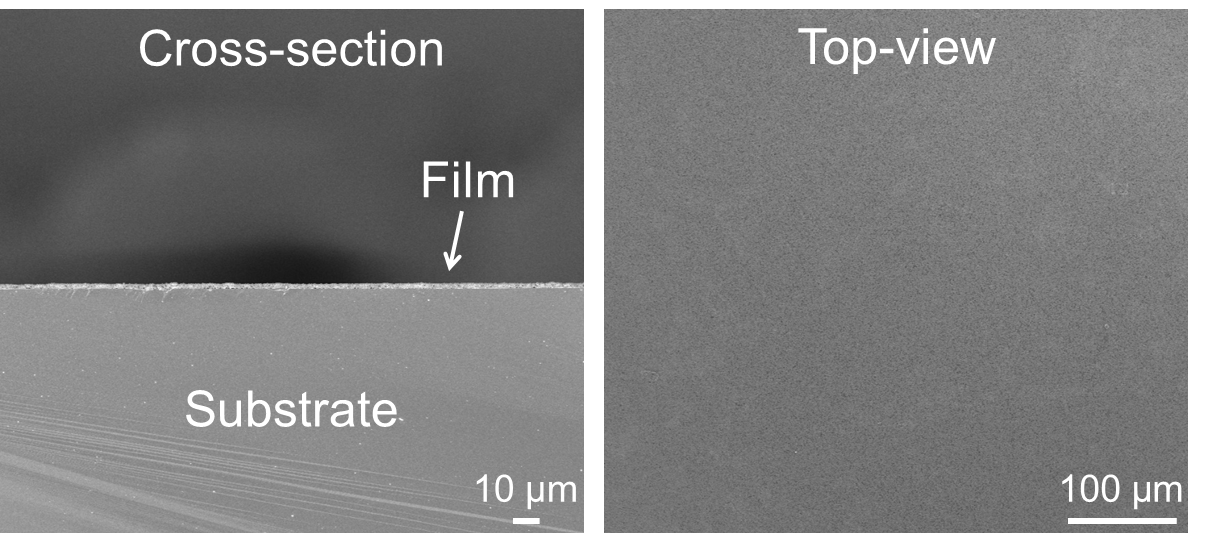
**

Figure S5. Low-magnification SEM images of the NP mixture [PS (diameter = 960 nm) and SiO_2_ (diameter = 700 nm) NPs, *α* ≈2] film obtained from the air-water (containing 0.8 wt % PEG) interface at the TA of 0.33.


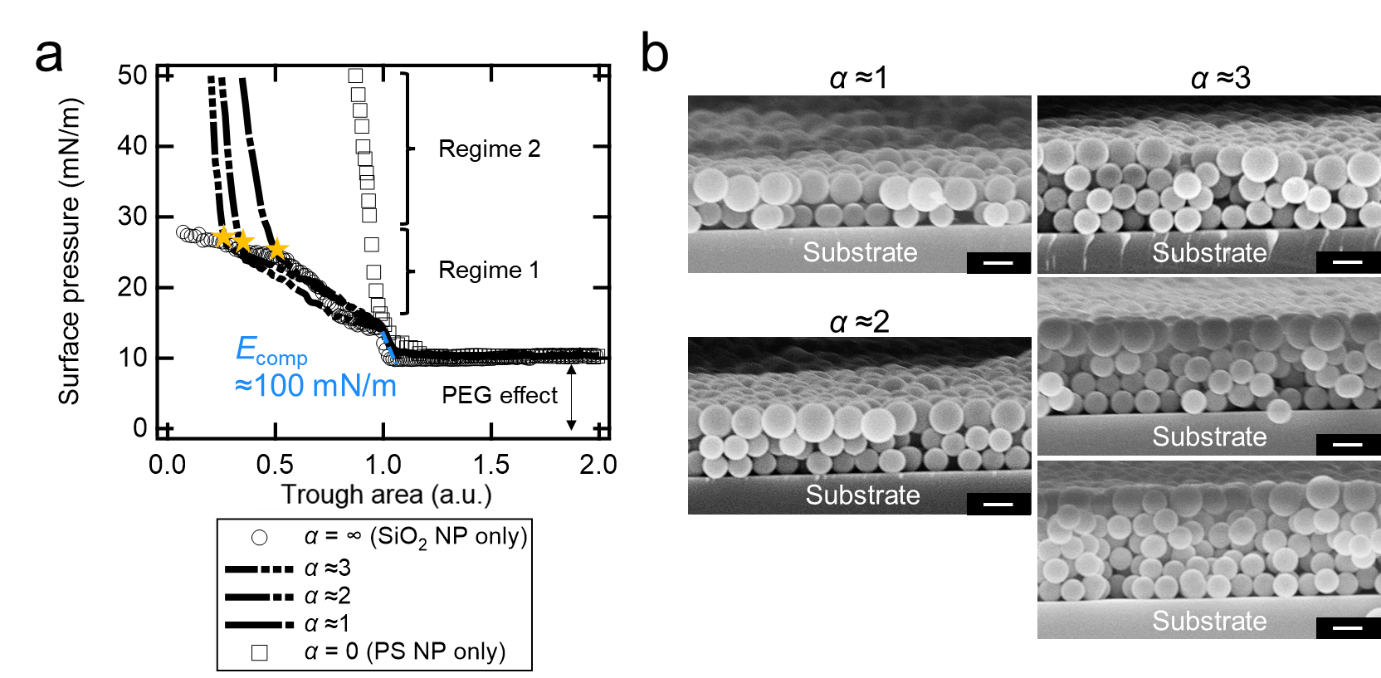


Figure S6. a) Compressional Langmuir isotherms of the NP mixtures [PS (diameter = 960 nm) and SiO_2_ (diameter = 700 nm)] at the air-water (containing 10^-2^ wt % PEG) interface according to the values of *α*. Detailed description can be analogously referred to that of Figure 2a. b) Cross-sectional SEM images of the stratified NP films deposited at the end of the regime 1 for each *α* value. Compared with the case of using the 0.8 wt % PEG solution, the stratified NP layers have inconsistent thicknesses at *α* ≈3, whereas the compositions of the stratified NP films are almost similar at *α* ≈1 and ≈2. All scale bars are 1 μm.


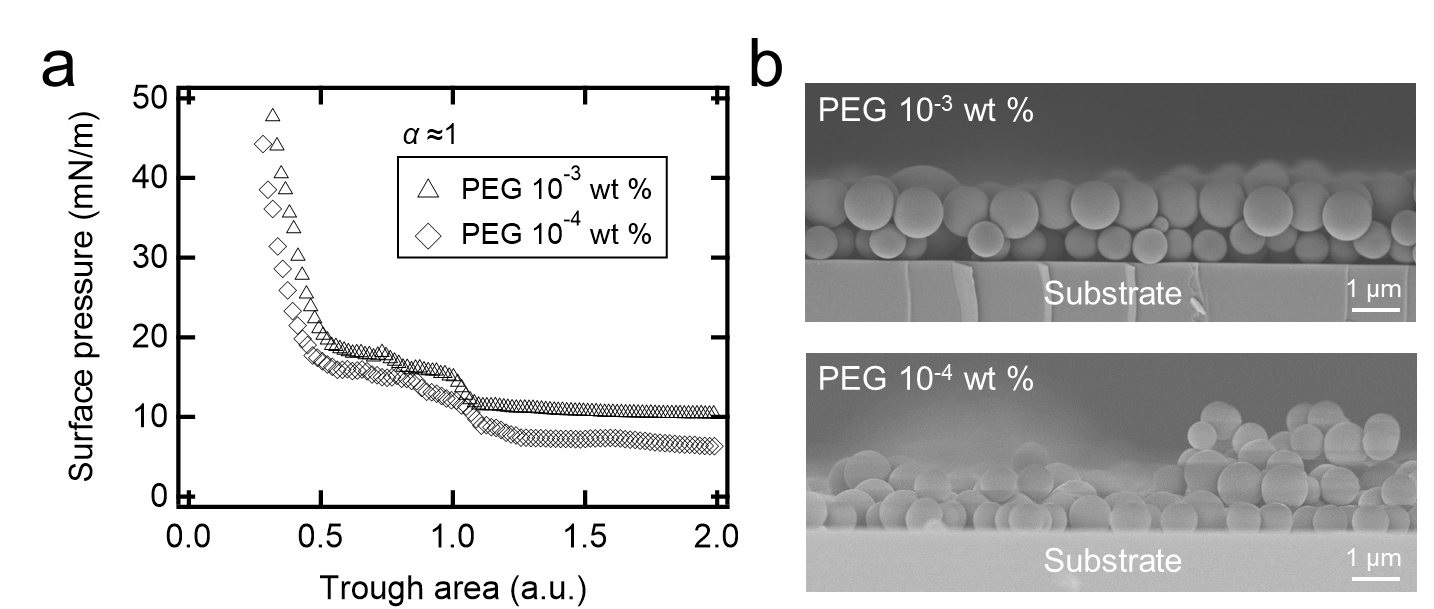


Figure S7. a) Compressional Langmuir isotherms of the NP mixtures [PS (diameter = 960 nm) and SiO_2_ (diameter = 700 nm)] (*α* ≈1) at the air-water (containing 10^-3^ and 10^-4^ wt % PEG) interfaces. Detailed description can be analogously referred to that of Figure 2a. b) Cross-sectional SEM images of the NP films deposited at the end of the regime 1 (trough area = 0.50). Stratification is successfully achieved at the PEG concentration of 10^-3^ wt %, whereas randomly thickened NP films are observed at the PEG concentration of 10^-4^ wt %. The discrepancy at the PEG concentration of 10^-4^ wt % is possibly because *P*_de_ is not able to suppress the desorption of the SiO_2_ NPs from the interface due to the vibration of the Langmuir trough.


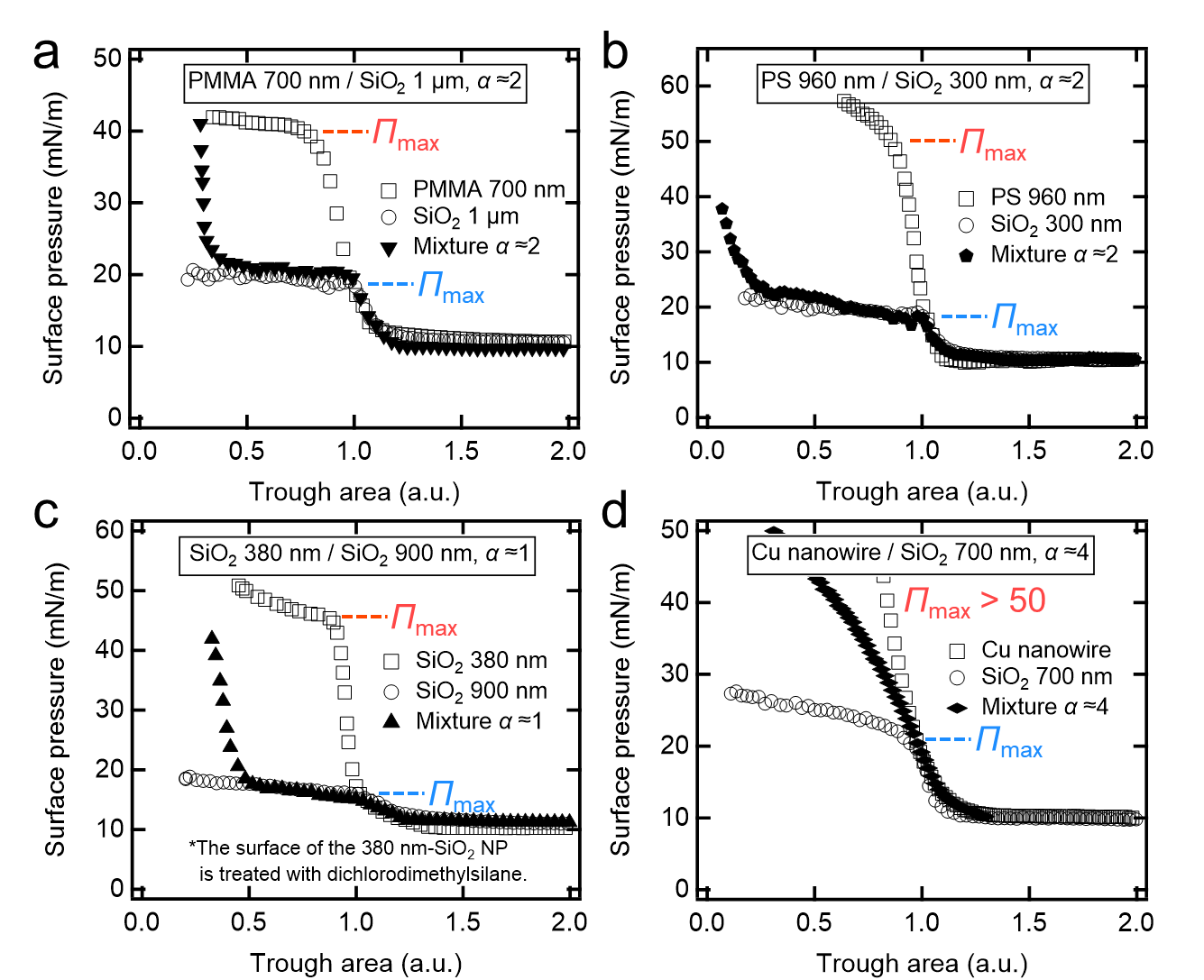


Figure S8. Compressional Langmuir isotherms of the NP mixture monolayers with various compositions at the air-water (containing 0.8 wt % PEG) interface. In (d), the isotherm of the collapsing mixture monolayer does not exhibit a combination of the regime 1 and 2; instead, it exhibits *Π* values between the isotherms of the Cu nanowire monolayer and the SiO_2_ NP monolayer. The structural rearrangement of the Cu nanowires, likely hindered by strong lateral capillary interactions between their end points,^[10]^ results in the inability to immediately release *Π* when the SiO_2_ NPs are desorbed.


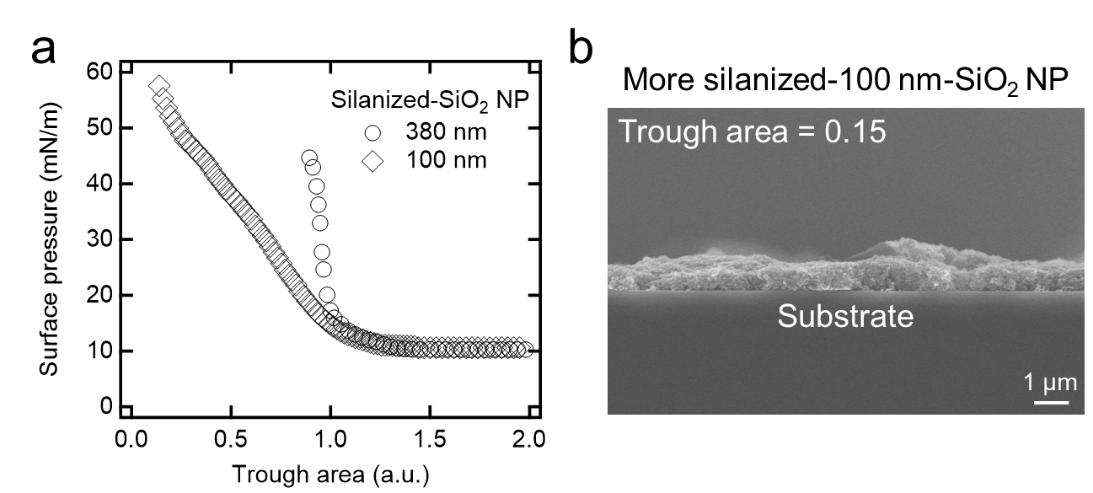


Figure S9. a) Compressional Langmuir isotherms of the silanized SiO_2_ NP monolayers at the air-water (containing 0.8 wt % PEG) interface. b) Cross-sectional SEM image of the more silanized-SiO_2_ NP (≈100 nm) film deposited at the trough area of 0.15.


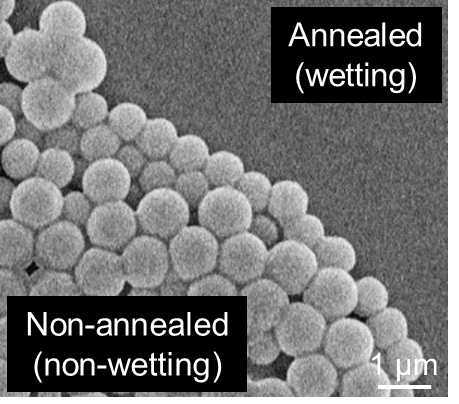


**Figure S10.** Top-view SEM image of the stratified NP [PS (diameter = 960 nm) and SiO_2_ (diameter = 700 nm)] film with *α* ≈2, partially annealed using a small amount of toluene. The annealed and non-annealed regions correspond to the wetting and non-wetting regions of toluene on the air-water interface.

References

[1] B. P. Binks, S. O. Lumsdon, *Langmuir* **2000**, *16*, 8622.

[2] J. Cui, Y. Ju, K. Liang, H. Ejima, S. Lörcher, K. T. Gause, J. J. Richardson, F. Caruso, *Soft Matter* **2014**, *10*, 2656.

[3] K. H. Kim, B. Q. Kim, J. Q. Kim, S. Q. Choi, *Journal of Physical Chemistry C* **2019**, *123*, 27862.

[4] S. Chakraborty, S. Ramakrishnan, *Langmuir* **2018**, *34*, 11729.

[5] L. Pocivavsek, R. Dellsy, A. Kern, S. Johnson, B. Lin, K. Y. C. Lee, E. Cerda, *Science* **2008**, *320*, 912.

[6] K. Kim, S. Kim, J. Ryu, J. Jeon, S. G. Jang, H. Kim, D. G. Gweon, W. Bin Im, Y. Han, H. Kim, S. Q. Choi, *Nat Commun* **2017**, *8*, 14305.

[7] P. L. Hansen, J. A. Cohen, R. Podgornik, V. A. Parsegian, *Biophys J* **2003**, *84*, 350.

[8] J. A. Cohen, R. Podgornik, P. L. Hansen, V. A. Parsegian, *Journal of Physical Chemistry B* **2009**, *113*, 3709.

[9] N. Shirahata, A. Hozumi, *J Nanosci Nanotechnol* **2006**, *6*, 1695.

[10] V. R. Dugyala, S. V. Daware, M. G. Basavaraj, *Soft Matter* **2013**, *9*, 6711.
